# Genome-Wide Identification and Analysis of Enhancer-Regulated microRNAs Across 31 Human Cancers

**DOI:** 10.3389/fgene.2020.00644

**Published:** 2020-06-30

**Authors:** Fei Tang, Yin Zhang, Qing-Qing Huang, Ming-Ming Qian, Zhi-Xue Li, Yan-Jing Li, Bei-Ping Li, Zheng-Liang Qiu, Jun-Jie Yue, Zhi-Yun Guo

**Affiliations:** ^1^School of Life Sciences and Engineering, Southwest Jiaotong University, Chengdu, China; ^2^Beijing Institute of Biotechnology, Beijing, China; ^3^Laboratory Animal Center, Academy of Military Medical Sciences, Beijing, China; ^4^Xinxiang Key Laboratory of Pathogenic Microbiology, Xinxiang, China

**Keywords:** enhancer, microRNA, cancer, transcriptional regulation, TCGA

## Abstract

Enhancers are *cis*-regulatory DNA elements that positively regulate the transcription of target genes in a tissue-specific manner, and dysregulation of target genes could lead to various diseases, such as cancer. Recent studies have shown that enhancers can regulate microRNAs (miRNAs) and participate in their biological synthesis. However, the network of enhancer-regulated miRNAs across multiple cancers is still unclear. Here, a total of 2,418 proximal enhancer–miRNA interactions and 1,280 distal enhancer–miRNA interactions were identified through the integration of genomic distance, co-expression, and 3D genome data in 31 cancers. The results showed that both proximal and distal interactions exhibited a significant cancer type-specific feature trend at the tissue level rather than at the single-cell level, and there was a noteworthy positive correlation between the expression of the miRNA and the number of enhancers regulating the same miRNA in most cancers. Furthermore, we found that there was a high correlation between the formation of enhancer–miRNA pairs and the expression of enhancer RNAs (eRNAs) whether in distal or proximal regulation. The characteristics analysis showed that miRes (enhancers that regulated miRNAs) and non-miRes presented significant differences in sequence conservation, guanine–cytosine (GC) content, and histone modification signatures. Notably, GC content, H3K4me1, and H3K36me3 were present differently between distal and proximal regulation, suggesting that they might participate in chromosome looping of enhancer–miRNA interactions. Finally, we introduced a case study, enhancer: chr1:1186391–1186507 ∼ miR-200a was highly relevant to the survival of thyroid cancer patients and a *cis*-eQTL SNP on the enhancer affected the expression of the TNFRSF18 gene as a tumor suppressor.

## Introduction

Enhancers are *cis*-regulatory DNA regulatory elements that positively regulate the transcription of target genes in a spatiotemporal-specific manner. The dysfunction of an enhancer has been considered to affect the enhancer–promoter communication and cause a lot of diseases, such as cancer (Thandapani, 2019). Previous studies have shown that enhancer activity is affected by the enhancer RNA (eRNA), which is transcribed bidirectionally from active regulatory enhancers and plays a key role in regulating downstream gene expression. The Functional Annotation of the Mammalian Genome (FANTOM) Group, which applied CAGE technology, had identified ∼65,000 active enhancers across multiple tissues, and these valuable resources provided important data sources for subsequent research (Andersson et al., 2014). Recently, a large-scale pan-cancer study for The Cancer Genome Atlas (TCGA) patient samples across 33 cancer types revealed that the enhancer activity affects the expression of a variety of tumor-associated genes and was involved in tumor tumorigenesis (Chen et al., 2018). MicroRNAs (miRNAs) are a subset of endogenous non-coding RNAs (∼22 nucleotides long) which play vital roles in regulating gene expression *via* targeting the specific sites in the 3′ untranslated region (3′-UTR) of mRNA (Lu and Rothenberg, 2018; Sandiford et al., 2018). In the past years, a great deal of literature confirmed that miRNAs are involved in almost all known cancers. A recent study showed that miR-24-1, which is present in the nucleus, promotes gene expression by targeting enhancers, suggesting that there is an obvious interaction between enhancers and miRNAs (Xiao et al., 2017). Other recent studies showed that enhancers (including typical enhancers and super enhancers) are found to regulate miRNA expression and participate in the biological synthesis of miRNAs regulated by Drosha/DGCR8 (Yun et al., 2018; Wood et al., 2019). These studies suggested that enhancers are involved in miRNA regulatory networks and contribute greatly to tumorigenesis and development.

However, the network of enhancer-regulated miRNAs across multiple tumors is still unclear. Therefore, a pan-cancer study was performed for enhancer-regulated miRNAs across the 33 human cancer types in TCGA. Based on the distance between the enhancer and the miRNA, enhancer–miRNA pairs were classified into two types: proximal and distal enhancer–miRNA regulation. A series of enhancer–miRNA regulations were identified through the integration of co-expression, distance information, 3D genome data of enhancers, and miRNAs from 8,693 samples in TCGA. Gene Ontology (GO) and Kyoto Encyclopedia of Genes and Genomes (KEGG) enrichment showed that the target genes of the enhancer-regulated miRNAs were significantly involved in tumor-associated biological processes and signaling pathways. Furthermore, we found that there was a high correlation between the formation of enhancer–miRNA pairs and the expression of eRNAs. The results showed that miRes (enhancers that regulated miRNAs) and non-miRes presented significantly different characteristics, including sequence conservation, guanine–cytosine (GC) content, and histone modification signatures. Several histone modifications revealed a significant cancer specificity and enhancer–miRNA spatial distance specificity. Finally, a case study was introduced, enhancer: chr1:1186391–1186507 ∼ miR-200a was highly relevant to the survival of thyroid cancer patients and the *cis*- expression quantitative trait loci (eQTL) SNP on the enhancer affected the expression of the TNFRSF18 gene as a tumor suppressor.

## Materials and Methods

### Identification of Enhancer–miRNA Interactions

Enhancer annotations and the expression data for 33 cancers from TCGA were downloaded from a previous study (Chen et al., 2018). The expression level of the enhancer in each sample is screened, and the enhancer with the expression level in 10% of the samples is considered to be an effectively expressed enhancer in the cancer. The expression data of miRNAs from 33 tumors and eight cancer adjacent normal tissues were downloaded from the TCGA database. The tumors corresponding to the eight adjacent normal tissues are lung adenocarcinoma (LUAD; 19 normal samples), liver hepatocellular carcinoma (LIHC; 50 normal samples), breast invasive carcinoma (BRCA; 104 normal samples), colon adenocarcinoma (COAD; 10 normal samples), head and neck squamous cell carcinoma (HNSC; 43 normal samples), prostate adenocarcinoma (PRAD; 52 normal samples), stomach adenocarcinoma (STAD; 31 normal samples), and thyroid carcinoma (THCA; 59 normal samples). Co-expression analysis of the enhancers and miRNAs was performed by using Spearman’s correlation analysis (correlation coefficient | *R*| > 0.1, *p* < 0.05). Co-expression analysis was performed on each cancer type, and we kept the number of samples with enhancer expression and samples with miRNA expression consistent. In addition, in order to eliminate the statistical impact caused by the difference in the overall sample size in each cancer, we further corrected them by following two methods: 4D genomic data and distance formula.

Based on the distance of the enhancer–miRNA interactions, they could be classified into two types: proximal regulation and distal regulation. Referring to a previous study (Suzuki et al., 2017), the linkage score *S* of proximal enhancer–miRNAs was calculated by the following formula: *S* (*B*/*A*) = (*M*-G)/(*M* + G). *M* and *G* represent the distance from the enhancer to the nearest miRNA gene and the nearest gene, respectively. The parameters *A* and *B* represent (G + M)/2^1/2^ and (*G*-M)/2^1/2^, respectively. According to the research of Suzuki, *S* < 0.2 was adopted as the threshold to screen the reliable enhancer–miRNA pairs. Distal enhancer–miRNA interactions were identified with the following procedure. Firstly, the transcription initiation sites (TSS) of 2,248 miRNAs were downloaded from the FANTOM5 data portal (Abugessaisa et al., 2017); 0.5 kb downstream and 1 kb upstream of the TSS of these miRNAs were defined as the putative promoter region. A total of 1,215 miRNAs were obtained through intersecting 1,881 miRNAs of TCGA and 2,248 miRNAs of FANTOM5. Human chromatin interaction data were downloaded from 4D Genome (Teng et al., 2016). If the enhancer and miRNA promoter regions overlap with the chromatin interaction region of the 4D Genome, it is considered that there is a physical interaction between the enhancer and the miRNA, and the pair is defined as distal regulation. According to previous researches, TAD is not always tissue-specific (Eagen, 2018). Therefore, we mapped the interaction of the enhancer–miRNAs obtained from the 4D Genome database in all tumor types.

### Cancer Type-Specific Enhancer–miRNA Interactions and Single-Cell Sequencing Data Analysis

Enhancer–miRNA interactions that present in one cancer were defined as cancer type-specific enhancer–miRNA interactions. The tissue corresponding to the cancer type specificity of genes was extracted from the database TissGDB (Kim et al., 2018). The single-cell sequencing data were obtained from the database CancerSEA (Yuan et al., 2019), and the gene expression levels are converted to TPM/CPM values. The expression value is subjected to log 2 conversion. T-SNE clustering was used to reveal the single-cell gene expression profiling.

### Identification and Analysis Ubiquitously Expressed Enhancer–miRNA Interactions

Enhancer–miRNA pairs that occurred in more than 10 cancer types were defined as ubiquitously expressed enhancer–miRNA interactions. In order to investigate the function of the miRNA involved in enhancer–miRNA interactions, we downloaded the experimentally confirmed miRNA target genes from the miRTarbase database. Furthermore, the target genes of each miRNA were subjected to GO and KEGG signaling pathway databases for functional enrichment analysis using R package clusterProfiler (Benjamini–Hochberg multiple tests, *p*_*adjust*_ < 0.05).

### Characteristics of Enhancer–miRNA Interactions

Enhancer RNAs were determined by aligning the RNA transcribed from the enhancer with the annotated RNA (GENCODE.v19). The transcripts overlapping the protein-coding genes were removed. The GC content data were downloaded from the UCSC GC Percent track (Haeussler et al., 2019). The GC content was taken as the average of the regions of the enhancer itself. The PhastCons score was obtained from the UCSC cons100way track (Siepel et al., 2005). The regions upstream and downstream which were 1 kb from the center of the enhancer were considered as the calculation range of conservation.

The nine obtainable histone modification CHIP-Seq data of eight cell lines were downloaded from the ENCODE, including H3K4me3, H3K4me1, H3K27ac, H3K9me3, H3K27me3, H3K36me3, H3K4me2, H3K9ac, and H2K20me1. The eight cell lines matched eight types of cancer: A549 (LUAD), HepG2 (LIHC), HELA (cervical squamous cell carcinoma, CESC), HCT116 (COAD), DOHH2 (diffuse large B cell lymphoma, DLBC), PC-3 (PRAD), PANC-1 (pancreatic adenocarcinoma, PAAD), and DND-41 (acute myeloid leukemia, LAML). The corresponding information comes from the database Expasy. The software bwtool is used to process the histone modification data of the enhancers (Pohl and Beato, 2014) and to obtain histone modification signals within 1 kb of the upstream and downstream regions of the enhancer central point. Signal consistency was considered when it appeared in at least five cancer types. Here, a point-biserial correlation test (correlation coefficient| rho| > 0.3, *q* < 0.05) and a *t* test (*p* < 0.05) were used to count whether miRes and non-miRes are different. The point-biserial correlation test is used to determine whether the difference of the histone signal is related to the type of enhancer, and the *t* test is used to judge the significance of this difference.

### eQTL and Survival Analysis

The eQTL data were retrieved from the PancanQTL database (Gong et al., 2018). A high correlation between the SNP located on the enhancers and the gene could be identified if the *q* value was lower than 0.05. Next, based on the database starBase (Li et al., 2014), the expression level of the target miRNA inferred for the enhancer in the disease was analyzed by patient survival.

## Results and Discussion

### Genome-Wide Identification of Enhancer–miRNA Interactions in 31 Cancers

**FIGURE 1:**
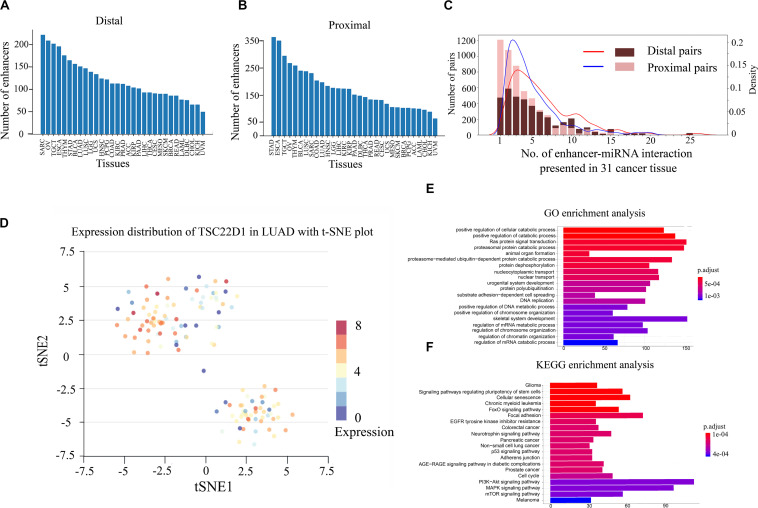
Gene Ontology (GO) and Kyoto Encyclopedia of Genes and Genomes (KEGG) pathway enrichment analyses and single-cell RNA sequencing (scRNA-seq) analysis of enhancer–miRNA regulations. Number of pairs of proximal **(A)** or distal **(B)** regulations present in each cancer type. **(C)** Frequency of occurrence of enhancer–miRNA interactions appearing in 31 cancers. **(D)** scRNA-seq analysis of the expression of TSC22D1 in lung adenocarcinoma (*LUAD*) using t-SNE cluster. GO **(E)** and KEGG pathway **(F)** enrichment analyses of the target genes of miRNAs that are regulated by ubiquitously expressed enhancers.

Previous studies have shown that enhancers are involved in the synthesis and regulation of miRNAs (Xiao et al., 2017). To further explore the mechanism of enhancer–miRNA regulation in cancers, we identified a series of enhancer-regulated miRNAs in 33 cancer types. The co-expression between 15,080 enhancers from 8,693 samples and 1,881 miRNAs in 33 cancers was first analyzed. Finally, all co-expression pairs of enhancer–miRNAs in 31 cancers were obtained, except for uterine corpus endometrial carcinoma (UCES), and glioblastoma multiforme (GBM) because of too few enhancers and miRNA samples in these two cancer types. Based on the distance between the enhancer and the miRNA, the enhancer–miRNA pairs were divided into two types: proximal and distal enhancer–miRNA regulation. For proximal regulation, the method presented in the previous study was used to calculate enhancer-regulated neighbor miRNAs (Suzuki et al., 2017). For distal enhancer–miRNA regulation, the enhancer–miRNA interactions were identified by Hi-C data from 4D Genome. As a result, a total of 2,418 proximal and 1,280 distal enhancer–miRNA pairs were obtained through the integration of genomic distance, co-expression, and interaction analysis in 31 cancers (Figures 1A,B and Supplementary Tables S1, S2). In addition, we counted sample information in all cancer types (including normal tissues corresponding to cancer) and obtained 348 distal and 553 proximal enhancer–miRNA pairs in eight cancer adjacent normal tissues (Supplementary Table S3). To investigate whether these enhancer–miRNA interactions were cancer type-specific or ubiquitously expressed, we counted the frequency of occurrence of these two types of interactions appearing in 31 cancers. The results revealed that both proximal and distal interactions exhibited a significant cancer type-specific feature (50.0% and 21.7% in proximal and distal interactions, respectively), with only a few number of regulations (1.2% and 2.5% in proximal and distal interactions, respectively) ubiquitously expressed (present in more than 10 cancers; Figure 1C). For example, miR-28 is regulated by two enhancers (chr3:187704282–187704692 and chr3:187686706–187686977), and this interaction only appears in LUAD across all 31 cancer types. Previous studies have shown that miR-28 plays a role in the negative associations of titanium with DNA damage in lung cancer (Chen et al., 2019). Furthermore, a previous study has shown that miR-28 can regulate TSC22D1, a gene specifically expressed in the LUAD (Kim et al., 2018). To investigate the heterogeneity of TSC22D1 in LUAD at the single-cell level, we performed single-cell sequencing cluster analysis by using t-SNE cluster (Figure 1D). The results revealed that this gene is widely expressed in various types of cells in LUAD, suggesting that the miRNA target gene is specific to tumors as part of the enhancer–miRNA regulatory network, but did not mean it was specific to single cells.

If the regulatory relationship appears across a large number of cancers, it suggests that these regulations are critical for the tumorigenesis and development. To explore the biological functions of these miRNAs which are involved in ubiquitously expressed enhancer–miRNA regulation, GO and KEGG functional enrichment analyses were performed using experimentally verified miRNA target genes. GO analysis indicated that these target genes of miRNAs that were regulated by ubiquitously expressed enhancers were significantly involved in tumor-associated biological processes such as cell cycle, cell differentiation, cell growth, metabolic regulation, metastasis, Ras protein catabolic process, etc., in distal (Figure 1E) or proximal regulation (Supplementary Figure S1A). KEGG analysis revealed that these miRNA target genes were significantly involved in cancer transcriptional dysregulation signaling pathways, such as FoxO signaling pathway, p53 signaling pathway, MAPK signaling pathway, the P13K-Akt signaling pathway, etc., in distal (Figure 1F) or proximal regulation (Supplementary Figure S1B).

### The Correlation Between the miRNA Expression and the Number of Enhancers That Regulate miRNAs

**FIGURE 2:**
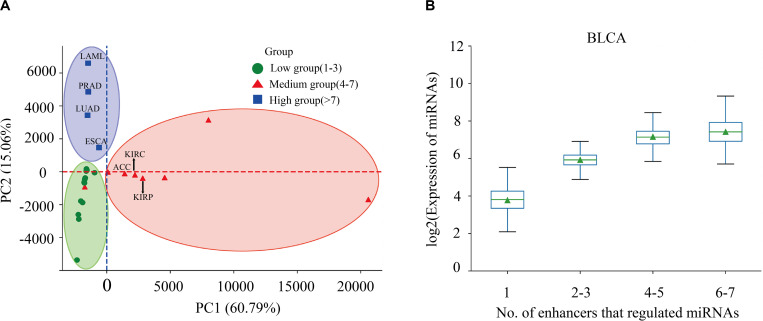
Correlation between miRNA expression and the number of regulating enhancers. **(A)** Principal component analysis (PCA) of the expression levels of miRNAs regulated by enhancers in 31 cancers. **(B)** Expressions of miRNAs that are regulated by different numbers of enhancers in bladder urothelial carcinoma (*BLCA*).

Enhancers often regulate target genes and do not strictly follow one-to-one regulatory relationships. In order to investigate whether there is a correlation between the expression level of miRNAs and the number of miRes regulating these miRNAs, we performed principal component analysis (PCA) of the expression levels of miRNAs regulated by enhancers in 31 cancers. Here, only the distal regulation was analyzed because most enhancer–miRNA interactions in proximal regulation followed a one-to-one regulatory rule according to the genomic position restriction. The PCA results showed that the 31 cancers could be divided into three groups according to the number of highly expressed miRNAs that were regulated by enhancers, as follows: low (one to three miRNAs), medium (four to seven miRNAs), and high (more than seven miRNAs; Figure 2A and Supplementary Table S4). A miRNA which has a higher expression than the median of miRNA expression is defined as a highly expressed miRNA. For example, the miRNAs in PRAD, LUAD, LAML, and esophageal carcinoma (ESCA) regulated by more than seven enhancers presented significantly higher expressions compared with the number of miRNAs regulated by enhancers that were less than seven (*p* < 0.05). Interestingly, some similar types of cancers tended to cluster into one group which shared the same enhancer–miRNA regulation pattern. For example, the most highly expressed miRNAs in three types of kidney cancers [adrenocortical carcinoma (ACC), kidney renal clear cell carcinoma (KIRC), and kidney renal papillary cell carcinoma (KIRP)] tended to be regulated by four to seven enhancers (Figure 2A). Notably, there was a significant positive correlation between the expression of miRNA and the number of enhancers that regulate miRNAs in bladder urothelial carcinoma (BLCA), lung squamous cell carcinoma (LUSC), ovarian serous cystadenocarcinoma (OV), and testicular germ cell tumors (TGCT; Figure 2B and Supplementary Figure S2).

### There Are Significant Differences in the Sequence Characteristics of miRes

**FIGURE 3:**
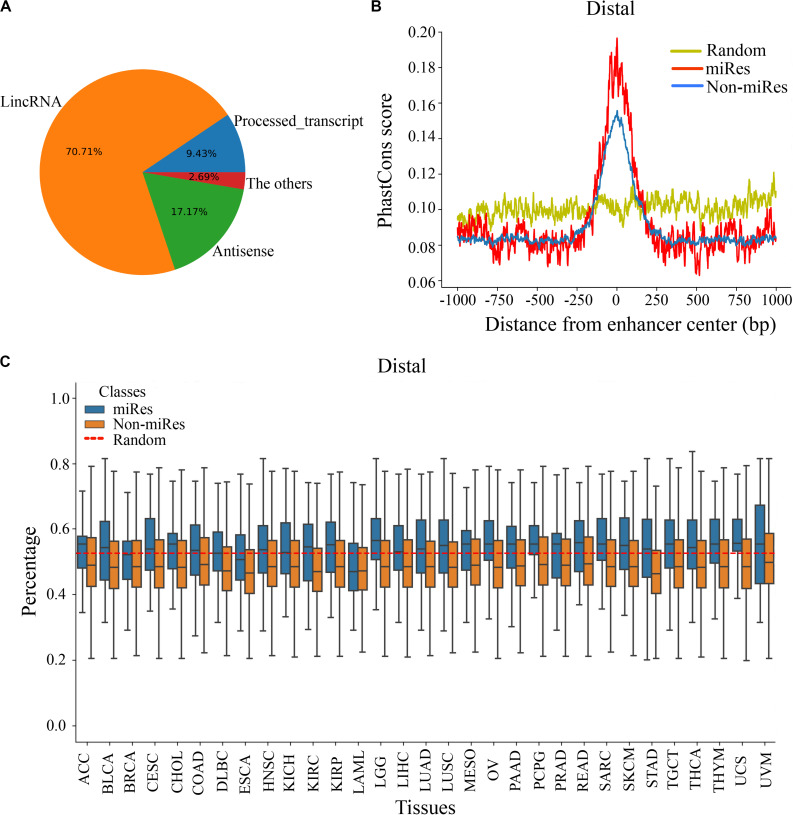
There are significant differences in the sequence characteristics of miRes. **(A)** Pie chart of all enhancer transcripts in distal regulation. **(B)** Conservative score of the enhancer sequence using PhastCons in distal regulation. **(C)** GC content of the enhancer in distal regulation.

It is important to explore the sequence characteristics of the miRes to conduct further identification of enhancer–miRNA interactions. Previously, it was reported that eRNA can be used as a *trans*-acting element to participate in the regulation of target genes (Zhang et al., 2019). Consequently, the human GENCODE annotation was first used to investigate the transcript types of the distal and proximal regulatory miRes. We found that 312 of the 998 (31.34%) enhancers that regulated distal miRNAs could transcribe known RNA species, and the largest proportion (70.71%) of RNAs was long intergenic non-coding RNAs (lincRNAs; Figure 3A). Similarly, the largest proportion of lincRNAs was also found present in enhancers that regulated proximal miRNAs (Supplementary Figure S3A). Moreover, we investigated whether there was a correlation between the formation of enhancer–miRNA pairs and the type of RNAs transcribed. The results showed that there was a high correlation between them in distal (chi-square test: *p* < 1.8e^–3^) and proximal regulation (chi-square test: *p* < 1e^–4^), which suggested that the enhancer might regulate the expression of miRNAs with the participation of eRNAs (Supplementary Tables S5, S6).

Continuously, PhastCons was used to analyze the conservation of the enhancer sequence. In distal regulation, the results showed that the sequence of the enhancer was more conservative than the random sequence (*p* < 3.2e^–23^), and the conserved region of the enhancer was mainly located within ±250 bp around its center (Figure 3B). Notably, the miRes showed higher conservation compared with the enhancers that did not regulate miRNAs. Similar results also appeared in proximal regulation (Supplementary Figure S3B). The above results indicated that the functional region of the enhancer mainly concentrated near the enhancer center and that the miRes exhibited greater conservation than did non-miRes. Furthermore, the GC contents of the distal and proximal regulatory miRes were calculated. The results showed that miRes exhibited significantly higher GC content than the average value of the random enhancer sequence in distal regulation (*p* < 2.6e^–22^) and the miRe exhibited a higher GC content than the non-miRe in each cancer type (Figure 3C). Interestingly, there was no significant difference between the GC contents of the miRes and non-miRes in proximal regulation (*p* > 0.05; Supplementary Figure S3C). Therefore, we speculated that the GC content was an inherent property of the enhancer and might have a potential impact on chromosome looping, which was more necessary in distal regulation than in proximal regulation.

### Histone Modification Showing Cancer- and miRes-Specific Features

**FIGURE 4:**
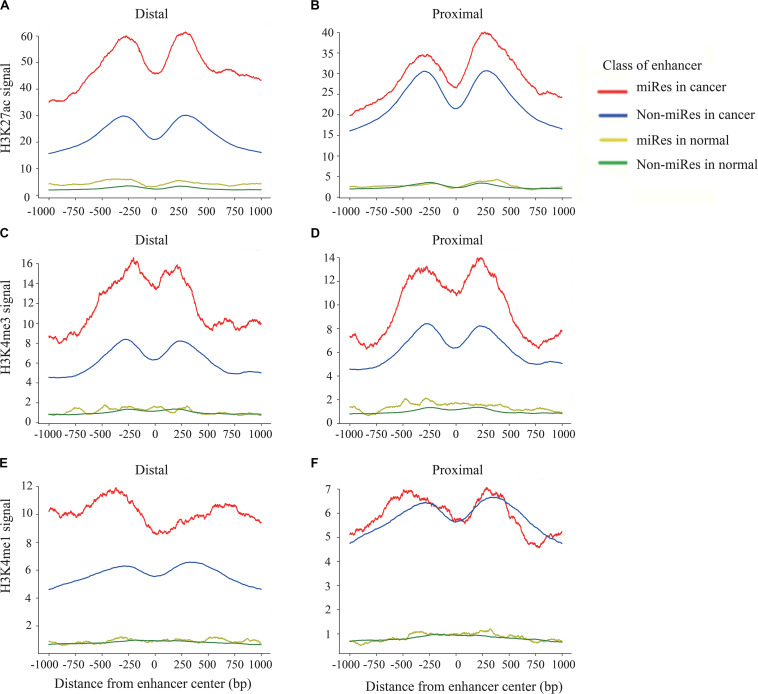
Signal of H3k27ac within ±1 kb surrounding the center of the enhancer in distal regulation **(A)** and proximal regulation **(B)**. Signal of H3k4me3 within ±1 kb surrounding the center of the enhancer in distal regulation **(C)** and proximal regulation **(D)**. Signal of H3k4me1 within ±1 kb surrounding the center of the enhancer in distal regulation **(E)** and proximal regulation **(F)**.

Previous studies have shown that the H3K27ac, H3K4me1, and H3K4me3 signals are key histone modification features for the activity of enhancers (Rada-Iglesias et al., 2012). To determine whether there are different activities between the miRes and non-miRes, we analyzed available H3K27ac, H3K4me1, and H3K4me3 ChIP-seq data from the ENCODE database in eight cancers using the software bwtool. Not surprisingly, as an example shown in Figures 4A–F, all of the enhancers in distal and proximal regulation pairs had an enrichment of H3K27ac, H3K4me3, and H3K4me1 signals in the range of 1 kb upstream and downstream from the center of the enhancer and presented significantly higher signals in cancers than in normal tissues. Notably, the signals of H3K27ac and H3K4me3 of the miRes were significantly higher than those of the non-miRes in most tumor tissues (Supplementary Figures S4–S7). Conversely, there was no significant difference in normal tissues. Interestingly, H3K4me1 showed that the difference between the miRe and non-miRe signals was only in distal regulation (Figure 4E and Supplementary Figure S8), but not in proximal regulation (Figure 4F and Supplementary Figure S9). This result was consistent with a previous study showing that enhancer activation of adjacent genes does not require H3K4me1 enrichment (Dorighi et al., 2017).

In addition, we asked whether there were other histone modifications in addition to the above signals that had a significant difference between the miRes and non-miRes. Therefore, we downloaded six histone modification data from ENCODE, including H3K4me2, H3K9ac, H3K20me1, H3K9me3, H3K27me3, and H3K36me3. We found that H3K9me3 and H3K36me3 in distal and proximal pairs were significantly different between the miRes and non-miRes in at least five out of the eight cancer tissues mentioned above (LUAD, LIHC, CESC, COAD, DLBC, PRAD, PAAD, and LAML; Supplementary Figures S10–S13). Among them, H3K9me3 showed lower enrichment in miRes compared with that in non-miRes, probably due to this histone modification which was the marker of heterochromatin (Supplementary Figures S10, S11; Becker et al., 2016). This result was consistent with our previous supposition that the transcription of enhancers had a positive effect on the expressions of miRNAs that are enhancer regulated. H3K36me3, a marker for transcription extension, showed a high enrichment in the miRes in distal interaction pairs, but not in the proximal interaction pairs. According to a previous study (Heinz et al., 2018), transcriptional elongation has an effect on the spatial structure of chromatin, and this may have more influence on distal regulation than on proximal regulation (Supplementary Figures S12, S13).

### A Case Study of miRe in Thyroid Carcinoma

**FIGURE 5:**
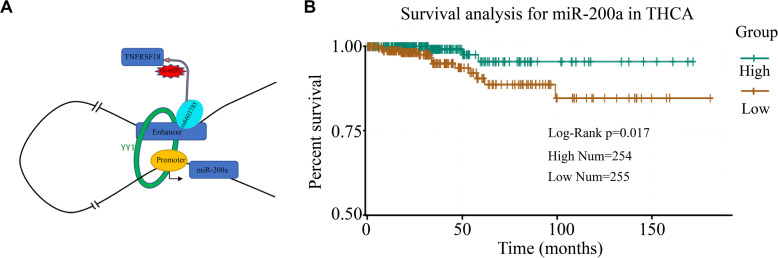
Case study of miRes in thyroid carcinoma. **(A)** An example of an enhancer–miRNA regulation interaction (chr1:1186391–1186507 ∼ miR-200a). **(B)** Survival curve of hsa-miR-200a in thyroid cancer.

To investigate the miRes identified as described above, here, we introduced a case study on an enhancer, chr1:1186391–1186507, and its target miRNA, miR-200a, in THCA. A *cis*-eQTL SNP (rs6603785) identified on enhancer chr1:1186391–1186507 is located close to the transcription start site (TSS) of the TNFRSF18 gene, which acts as a tumor suppressor (Xiong et al., 2019), and mainly occurs when the base A mutates to T (Figure 5A). YY1 in Figure 5A is an important transcription factor clearly associated with chromatin looping (Weintraub et al., 2017). It can be used as an enhancer–promoter loop structure regulator. There was a significant difference in the expression levels of samples of different genotypes (*F* test: *p* < 1.76e^–4^; Supplementary Figure S14). In addition, miR-200a, as a target of the enhancer, was highly relevant to the survival of thyroid cancer patients (Figure 5B). A previous study has shown that miR-200a regulates the epithelial stromal transformation of thyroid cancer through the EGF/EGFR signal (Xue et al., 2015), and it is a key factor in the epithelial phenotype and a tumor suppressor in THCA (Wang et al., 2018). Additionally, the survival analysis showed that patients with low expression of miR-200a have a lower survival time.

## Conclusion

We identify a series of enhancer–miRNA regulations in 31 human cancers. The results showed that enhancer–miRNA interactions exhibited a significant cancer type-specific feature and a high correlation between the formation of the enhancer–miRNA pairs and the expression of the eRNAs. The characteristics analysis demonstrated that the miRes and non-miRes presented significant differences in sequence conservation, GC content, and histone modification signatures. Notably, GC content, H3K4me1, and H3K36me3 were revealed as significantly different signals in distal and proximal regulation. Finally, we introduced a case study, enhancer: chr1:1186391–1186507 ∼ miR-200a was highly relevant to the survival of thyroid cancer patients and a *cis*-eQTL SNP on enhancer affected the expression of the TNFRSF18 gene, a tumor suppressor.

## Data Availability Statement

The datasets generated for this study can be found in the TCGA (https://portal.gdc.cancer.gov/), FANTOM5 (https://fantom.org/), and ENCODE (https://www.encodeproject.org/).

## Author Contributions

FT, Y-JL, and Z-YG formulated the study. FT, M-MQ, and Q-QH were responsible for the acquisition of data. FT, Z-XL, and YZ analyzed the data. Z-YG, Z-LQ, B-PL, and Q-QH participated in the analysis, discussion, and support. FT, Y-JL, and Z-YG drafted the manuscript. FT, Z-YG, and J-JY revised the manuscript. All authors contributed to the article and approved the submitted version.

## Conflict of Interest

The authors declare that the research was conducted in the absence of any commercial or financial relationships that could be construed as a potential conflict of interest.

## Supplementary Material

FIGURE S1 Pathway enrichment analysis of proximal enhancer-miRNA regulation pairs. **(A)** GO enrichment analysis of target genes of miRNAs that were regulated by ubiquitously expressed enhancers in proximal regulation pairs. Same as distal regulation pairs, GO analysis indicated that these target genes of miRNAs that were regulated by ubiquitously expressed enhancers were significantly involved in tumor-associated biological processes. **(B)** KEGG enrichment analysis of target genes of miRNAs that were regulated by ubiquitously expressed enhancers in proximal regulation pairs. Same as distal regulation pairs, these target genes of miRNA in proximal regulation pairs were significantly involved in cancer transcriptional dysregulation signaling pathways.

FIGURE S2 Correlation between number of contraction enhancers and miRNA expression. There was a significant positive correlation between the expression of miRNA and the number of regulating enhancers in the Testicular Germ Cell Tumors (TGCT), Ovarian serous cystadenocarcinoma (OV), and Lung squamous cell carcinoma (LUSC).

FIGURE S3 Sequence characteristics of miRes and non-miRes in proximal regulation. **(A)** Pie chart of all enhancer transcriptions in proximal regulation. 973 of the 2418 (31.34%) enhancers that regulated distal miRNAs could transcribe eRNA, and the largest proportion (61.18%) of RNAs was lincRNA. **(B)** Conservative score of enhancers in proximal regulation. The results showed that the sequence of the enhancer was more conservative than the random sequence (*p* < 7.0e-28), and the miRes showed a higher conservation compared with the enhancers that did not regulate miRNAs. **(C)** GC content of the enhancer in proximal regulation. Unlike distal regulation pairs, there was no significant difference between the GC content of miRes and non-miRes in proximal regulation (*P* > 0.05).

FIGURE S4 The signal of H3K27ac within ±1 kb of the enhancer center in distal regulation pairs. The signals of H3K27ac of miRes in distal regulation pairs were significantly higher than those of non-miRes in most tumor tissues. There was no significant difference in normal tissues. Except for PAAD, the signal of enhancer in cancer tissue was higher than that of enhancer in normal tissue in most diseases.

FIGURE S5 The signal of H3K27ac within ±1 kb of the enhancer center in proximal regulation pairs. The signals of H3K27ac of miRes in proximal regulation pairs were significantly higher than those of non-miRes in most tumor tissues. There was no significant difference in normal tissues. Except for COAD and PAAD, the signal of enhancer in cancer tissue was higher than that of enhancer in normal tissue in most diseases.

FIGURE S6 The signal of H3K4me3 within ±1 kb of the enhancer center in distal regulation pairs. The signals of H3K4me3 of miRes in distal regulation pairs were significantly higher than those of non-miRes in most tumor tissues, and the signal of enhancer in cancer tissue was higher than that of enhancer in normal tissue in most diseases.

FIGURE S7 The signal of H3K4me3 within ±1 kb of the enhancer center in proximal regulation pairs. The signals of H3K4me3 of miRes in proximal regulation pairs were significantly higher than those of non-miRes in most tumor tissues. There was no significant difference in normal tissues. Except for COAD, the signal of enhancer in cancer tissue was higher than that of enhancer in normal tissue in most diseases.

FIGURE S8 The signal of H3K4me1 within ±1 kb of the enhancer center in distal regulation pairs. The signals of H3K4me1 of miRes in distal regulation pairs were significantly higher than those of non-miRes in most tumor tissues. There was no significant difference in normal tissues. Expect for PAAD, the signal of enhancer in cancer tissue was higher than that of enhancer in normal tissue in most diseases.

FIGURE S9 The signal of H3K4me1 within ±1 kb of the enhancer center in proximal regulation pairs. There was no significant difference between the H3K4me3 signal of miRes in proximal regulation pairs and that of non-miRes in most tumor tissues. There was no significant difference in normal tissues. Except for PAAD, the signal of enhancer in cancer tissue was higher than that of enhancer in normal tissue in most diseases.

FIGURE S10 The signal of H3K9me3 within ±1 kb of the enhancer center in distal regulation pairs. The signal of H3K9me3 showed lower enrichment in miRes of distal regulation pairs compared with in non-miRes in most tumor tissues. There was no significant difference in normal tissues.

FIGURE S11 The signal of H3K9me3 within ±1 kb of the enhancer center in proximal regulation pairs. The signal of H3K9me3 showed lower enrichment in miRes of proximal regulation pairs compared with in non-miRes in most tumor tissues. There was no significant difference in normal tissues.

FIGURE S12 The signal of H3K36me3 within ±1 kb of the enhancer center in distal regulation pairs. The signal of H3K36me3 showed higher enrichment in miRes of distal regulation pairs compared with in non-miRes in most tumor tissues. There was no significant difference in normal tissues.

FIGURE S13 The signal of H3K36me3 within ±1 kb of the enhancer center in proximal regulation pairs. The signal of H3k36me3 had no significant difference in the miRe of distal regulation pairs in most tumor and normal tissues compared with that of non-miRe.

FIGURE S14 Expression of three genotypes of rs6603785. Significant differences in the expression levels of the three genotypes of this SNP (rs6603785).

TABLE S1 Identified 1,280 distal enhancer-miRNA regulation pairs in 31 cancer types.

TABLE S2 Identified 2,418 proximal enhancer-miRNA regulation pairs in 31 cancer types.

TABLE S3 Identified 554 proximal enhancer-miRNA regulation pairs, 348 distal enhancer-miRNA regulation pairs in 8 cancer adjacent normal tissues and sample information of all types of cancer.

TABLE S4 PCA of the expression levels of miRNAs regulated by enhancers in 31 cancer types.

TABLE S5 Enhancers regulating miRNA associated with known transcription of enhancers in distal regulation.

TABLE S6 Enhancers regulating miRNA associated with known transcription of enhancers in proximal regulation.
