# Supplementary material for: Genome-Wide Identification and Analysis of Enhancer-Regulated microRNAs Across 31 Human Cancers
Source: Front Genet. 2020 Jun 30;11:644. doi: 10.3389/fgene.2020.00644 (PMC7344161; doi:10.3389/fgene.2020.00644)
Supplement: TABLE S4 — PCA of the expression levels of miRNAs regulated by enhancers in 31 cancer types. [file Table_4.PDF]

Table S4. PCA of the expression levels of miRNAs regulated by enhancers in 31 cancer types

Group	Cancer type
High group（>7）	ESCA，LAML，LUAD，PRAD
Medium group（4-5）	ACC, BRCA, COAD, KIRC, KIRP, PAAD, SARC
Low group（1-3）	CESC，CHOL，DLBC，HNSC，KICH，LGG，LIHC，MESO，PCPG，READ，SKCM，STAD，THCA，THYM，UCS，UVM，BLCA，LUSC，TGCT，OV

Ps: Red diseases indicate the group with positive correlation.
