# Supplementary material for: Genome-Wide Identification and Analysis of Enhancer-Regulated microRNAs Across 31 Human Cancers
Source: Front Genet. 2020 Jun 30;11:644. doi: 10.3389/fgene.2020.00644 (PMC7344161; doi:10.3389/fgene.2020.00644)
Supplement: TABLE S5 — Enhancers regulating miRNA associated with known transcription of enhancers in distal regulation. [file Table_5.PDF]

**Table S5.** Enhancers regulating miRNA associated with known transcription of enhancers in distal regulation

| Number | miRes | Non-miRes | Total |
|---|---|---|---|
| Enhancer with known transcript | 321 | 4109 | 4430 |
| Enhancer without known transcripts | 686 | 10692 | 11378 |
| Total | 1007 | 14801 | 15808 |

Chi square-value >7.71 P-value <$5.48e^{-4}$
