# Supplementary material for: Genome-Wide Identification and Analysis of Enhancer-Regulated microRNAs Across 31 Human Cancers
Source: Front Genet. 2020 Jun 30;11:644. doi: 10.3389/fgene.2020.00644 (PMC7344161; doi:10.3389/fgene.2020.00644)
Supplement: TABLE S6 — Enhancers regulating miRNA associated with known transcription of enhancers in proximal regulation. [file Table_6.PDF]

Table S6. Enhancers regulating miRNA associated with known transcription of enhancers in proximal regulation

Number	miRes	Non-miRes	Total
Enhancer with known transcripts	973	3448	4421
Enhancer without known transcripts	1445	9942	11387
Total	2418	13390	15808

Chi square-value >212.72 P-value $<3.5e^{-47}$
